# Effectiveness of Pfizer-BioNTech and Moderna Vaccines Against COVID-19 Among Hospitalized Adults Aged ≥65 Years — United States, January–March 2021

**DOI:** 10.15585/mmwr.mm7018e1

**Published:** 2021-05-07

**Authors:** Mark W. Tenforde, Samantha M. Olson, Wesley H. Self, H. Keipp Talbot, Christopher J. Lindsell, Jay S. Steingrub, Nathan I. Shapiro, Adit A. Ginde, David J. Douin, Matthew E. Prekker, Samuel M. Brown, Ithan D. Peltan, Michelle N. Gong, Amira Mohamed, Akram Khan, Matthew C. Exline, D. Clark Files, Kevin W. Gibbs, William B. Stubblefield, Jonathan D. Casey, Todd W. Rice, Carlos G. Grijalva, David N. Hager, Arber Shehu, Nida Qadir, Steven Y. Chang, Jennifer G. Wilson, Manjusha Gaglani, Kempapura Murthy, Nicole Calhoun, Arnold S. Monto, Emily T. Martin, Anurag Malani, Richard K. Zimmerman, Fernanda P. Silveira, Donald B. Middleton, Yuwei Zhu, Dayna Wyatt, Meagan Stephenson, Adrienne Baughman, Kelsey N. Womack, Kimberly W. Hart, Miwako Kobayashi, Jennifer R. Verani, Manish M. Patel, IVY Network, HAIVEN Investigators, IVY Network, Omowunmi Amosu, Brent Armbruster, Valerie Aston, Marianne Bernardo, Robert Bowers, Leslie De Souza, Jennifer Friedel, Kevin Gardner, Jennifer Goff, Alexandra June Gordon, Audrey Hendrickson, Madeline Hicks, Michelle Howell, Jakea Johnson, Jeffrey Jorgensen, Sarah Karow, Lori Kozikowski, Olivia Krol, Leigha Landreth, Mary LaRose, Brenda Lopez, New York, Andrea Luong, Bob McClellan, Ellen Maruggi, Karen Miller, Rahul Nair, Lisa Parks, Jennifer Peers, Cynthia Perez, Adreanne Rivera, Jonasel Roque, Andres Santana, Tyler Scharber, Emma Silverman, Michael Tozier, Hiwet Tzehaie, Zachary Zouyed, HAIVEN Investigators, Alejandro Arroliga, Alicia Bagiatis, GK Balasubramani, Caroline K. Cheng, Heather Eng, Shekhar Ghamande, Judy Herrick, Eric Hoffman, Kailey Hughes, Lois E. Lamerato, Adam S. Lauring, Amanda McKillop, Tresa McNeal, E.J. McSpadden, John Midturi, Manohar Mutnal, Mary Patricia Nowalk, Joshua G. Petrie, Chandni Raiyani, Arundhati Rao, Sean G. Saul, Theresa M. Sax, Hannah E. Segaloff, Lori Stiefel, Marcus Volz, Kimberly Walker, Nicole Wheeler, Heath White, John V. Williams, Mohamed Yassin, Martha Zayed, Tnelda Zunie

**Affiliations:** ^1^CDC COVID-19 Response Team; ^2^Vanderbilt University Medical Center, Nashville, Tennessee; ^3^Baystate Medical Center, Springfield, Massachusetts; ^4^Beth Israel Deaconess Medical Center, Boston, Massachusetts; ^5^University of Colorado School of Medicine, Aurora, Colorado; ^6^Hennepin County Medical Center, Minneapolis, Minnesota; ^7^Intermountain Medical Center and University of Utah, Salt Lake City, Utah; ^8^Montefiore Healthcare Center, Albert Einstein College of Medicine, Bronx, New York; ^9^Oregon Health & Science University Hospital, Portland, Oregon; ^10^Ohio State University Wexner Medical Center, Columbus, Ohio; ^11^Wake Forest University Baptist Medical Center, Winston-Salem, North Carolina; ^12^Johns Hopkins Hospital, Baltimore, Maryland; ^13^Ronald Reagan-UCLA Medical Center, Los Angeles, California; ^14^Stanford University School of Medicine, Palo Alto, California; ^15^Baylor Scott & White Health, Temple, Texas; ^16^Texas A&M University College of Medicine, Temple, Texas; ^17^University of Michigan School of Public Health, Ann Arbor, Michigan; ^18^St. Joseph Mercy Health System, Ann Arbor, Michigan; ^19^University of Pittsburgh Schools of the Health Sciences, University of Pittsburgh Medical Center, Pittsburgh, Pennsylvania.; Montefiore Healthcare Center, Albert Einstein College of Medicine, Bronx, New York; Intermountain Medical Center, Salt Lake City, Utah; Intermountain Medical Center, Salt Lake City, Utah; Reagan-UCLA Medical Center, Los Angeles, California; Intermountain Medical Center, Salt Lake City, Utah; Baystate Medical Center, Springfield, Massachusetts; University of Colorado School of Medicine, Aurora, Colorado; Stanford University School of Medicine, Palo Alto, California; University of Colorado School of Medicine, Aurora, Colorado; Stanford University School of Medicine, Palo Alto, California; Hennepin County Medical Center, Minneapolis, Minnesota; Wake Forest University Baptist Medical Center, Winston-Salem, North Carolina; University of Colorado School of Medicine, Aurora, Colorado; Vanderbilt University Medical Center, Nashville, Tennessee; Intermountain Medical Center, Salt Lake City, Utah; Ohio State University Wexner Medical Center, Columbus, Ohio; Baystate Medical Center, Springfield, Massachusetts; Oregon Health & Science University, Portland, Oregon; Wake Forest University Baptist Medical Center, Winston-Salem, North Carolina; Wake Forest University Baptist Medical Center, Winston-Salem, North Carolina; Montefiore Healthcare Center; Albert Einstein College of Medicine, Bronx; Oregon Health & Science University, Portland, Oregon; Vanderbilt University Medical Center, Nashville, Tennessee; Hennepin County Medical Center, Minneapolis, Minnesota; Vanderbilt University Medical Center, Nashville, Tennessee; Montefiore Healthcare Center, Albert Einstein College of Medicine, Bronx, New York; Wake Forest University Baptist Medical Center, Winston-Salem, North Carolina; University of Colorado School of Medicine, Aurora, Colorado; Stanford University School of Medicine, Palo Alto, California; Reagan-UCLA Medical Center, Los Angeles, California; Stanford University School of Medicine, Palo Alto, California; Baystate Medical Center, Springfield, Massachusetts; Hennepin County Medical Center, Minneapolis, Minnesota; Oregon Health & Science University, Portland, Oregon; University of Colorado School of Medicine, Aurora, Colorado; Montefiore Healthcare Center, Albert Einstein College of Medicine, Bronx, New York; Oregon Health & Science University, Portland, Oregon; Baylor Scott & White Health, Texas A&M University College of Medicine, Temple, Texas; University of Pittsburgh Schools of the Health Sciences, Pittsburgh, Pennsylvania; University of Pittsburgh Schools of the Health Sciences, Pittsburgh, Pennsylvania; University of Michigan School of Public Health, Ann Arbor, Michigan; University of Pittsburgh Schools of the Health Sciences, Pittsburgh, Pennsylvania; Baylor Scott & White Health, Texas A&M University College of Medicine, Temple, Texas; Baylor Scott & White Health, Temple, Texas; Baylor Scott & White Health, Temple, Texas; University of Pittsburgh Schools of the Health Sciences, University of Pittsburgh Medical Center, Pittsburgh, Pennsylvania; Henry Ford Health System, Detroit, Michigan; University of Michigan School of Public Health, Ann Arbor, Michigan; Baylor Scott & White Health, Temple, Texas; Baylor Scott & White Health, Texas A&M University College of Medicine, Temple, Texas; University of Michigan School of Public Health, Ann Arbor, Michigan; Baylor Scott & White Health, Texas A&M University College of Medicine, Temple, Texas; Baylor Scott & White Health, Texas A&M University College of Medicine, Temple, Texas; University of Pittsburgh Schools of the Health Sciences, Pittsburgh, Pennsylvania; University of Michigan School of Public Health, Ann Arbor, Michigan; Baylor Scott & White Health, Temple, Texas; Baylor Scott & White Health, Texas A&M University College of Medicine, Temple, Texas; University of Pittsburgh Schools of the Health Sciences, Pittsburgh, Pennsylvania; University of Pittsburgh Schools of the Health Sciences, Pittsburgh, Pennsylvania; University of Michigan School of Public Health, Ann Arbor, Michigan; University of Pittsburgh Schools of the Health Sciences, University of Pittsburgh Medical Center, Pittsburgh, Pennsylvania; Baylor Scott & White Health, Temple, Texas; Baylor Scott & White Health, Temple, Texas; University of Pittsburgh Medical Center, Pittsburgh, Pennsylvania; Baylor Scott & White Health, Texas A&M University College of Medicine, Temple, Texas; University of Pittsburgh Schools of the Health Sciences, University of Pittsburgh Medical Center, Pittsburgh, Pennsylvania; University of Pittsburgh Schools of the Health Sciences, University of Pittsburgh Medical Center, Pittsburgh, Pennsylvania; Baylor Scott & White Health, Temple, Texas; Baylor Scott & White Health, Temple, Texas

Adults aged ≥65 years are at increased risk for severe outcomes from COVID-19 and were identified as a priority group to receive the first COVID-19 vaccines approved for use under an Emergency Use Authorization (EUA) in the United States (*1*–*3*). In an evaluation at 24 hospitals in 14 states,* the effectiveness of partial or full vaccination^†^ with Pfizer-BioNTech or Moderna vaccines against COVID-19–associated hospitalization was assessed among adults aged ≥65 years. Among 417 hospitalized adults aged ≥65 years (including 187 case-patients and 230 controls), the median age was 73 years, 48% were female, 73% were non-Hispanic White, 17% were non-Hispanic Black, 6% were Hispanic, and 4% lived in a long-term care facility. Adjusted vaccine effectiveness (VE) against COVID-19–associated hospitalization among adults aged ≥65 years was estimated to be 94% (95% confidence interval [CI] = 49%–99%) for full vaccination and 64% (95% CI = 28%–82%) for partial vaccination. These findings are consistent with efficacy determined from clinical trials in the subgroup of adults aged ≥65 years (*4*,*5*). This multisite U.S. evaluation under real-world conditions suggests that vaccination provided protection against COVID-19–associated hospitalization among adults aged ≥65 years. Vaccination is a critical tool for reducing severe COVID-19 in groups at high risk.

Randomized clinical trials of vaccines that have received an EUA in the United States showed efficacy of 94%–95% in preventing COVID-19–associated illness (*4*,*5*).^§^ However, hospitalization is a rare outcome among patients with COVID-19–associated illness of any severity, so most cases detected in the trials did not lead to hospitalization; therefore, the studies had limited power to assess protection against severe COVID-19 among older adults. Postmarketing observational studies are important to assess VE against COVID-19–associated hospitalizations in adults aged ≥65 years under real-world conditions and to strengthen evidence from clinical trials of vaccine efficacy. A standard approach to postmarketing VE evaluation involves the test-negative design in which vaccine performance is assessed by comparing the odds of antecedent vaccination among case-patients with acute laboratory-confirmed COVID-19 and control-patients without acute COVID-19 (*6*).

During January 1, 2021–March 26, 2021, adults with COVID-19–like illness^¶^ admitted to 24 hospitals in 14 states within two networks (the Hospitalized Adult Influenza Vaccine Effectiveness Network [HAIVEN] and the Influenza and Other Viruses in the Acutely Ill [IVY] Network) were enrolled. Patients were eligible if they were aged ≥65 years on the date of hospital admission, received clinical testing for SARS-CoV-2 (the virus that causes COVID-19) by reverse transcription–polymerase chain reaction (RT-PCR) or antigen test within 10 days of illness onset, and had onset of symptoms 0–14 days before admission. Case-patients were those who received one or more positive test results for SARS-CoV-2. Patients meeting eligibility criteria who received negative SARS-CoV-2 RT-PCR test results served as controls. Baseline demographic and health information, details about the current illness, and SARS-CoV-2 testing history were obtained by patient or proxy interviews with trained study personnel and electronic medical record review. Patients or proxies were asked about SARS-CoV-2 vaccination history including number of doses, dates and location of vaccination, and availability of vaccination record cards documenting receipt. Secondary electronic medical records and state immunization registry searches for SARS-CoV-2 vaccination records were conducted during March 26, 2021–April 19, 2021, for all included patients without vaccination record cards to verify reported or unknown vaccination status.

Participants were considered to have received COVID-19 vaccine doses based on documentation by CDC vaccination record card, state immunization registry search, electronic medical record search, or by plausible self-report if they provided vaccination dates and location. Documented record of vaccination dates was used when any potential discordance was identified between self-reported and documented dates. Participants with unverified COVID-19 testing status or vaccination status, or vaccination with Janssen COVID-19 vaccine (Johnson & Johnson), which was in limited use during the evaluation period, were not included. SARS-CoV-2 vaccination status included four categories: 1) unvaccinated, defined as no receipt of any SARS CoV-2 vaccine before illness onset; 2) single-dose vaccinated <14 days before illness, defined as receipt of the first vaccine dose <14 days before COVID-19–like illness onset; 3) partially vaccinated, defined as receipt of 1 dose of a 2-dose vaccination series (Pfizer-BioNTech or Moderna vaccines) ≥14 days before illness onset or 2 doses, with the second dose received <14 days before illness onset** (*7*); and 4) fully vaccinated, defined as receipt of both doses of a 2-dose vaccine series, with the second dose received ≥14 days before illness onset. Estimates of VE were calculated by comparing the odds of SARS-CoV-2 vaccination in case-patients and controls using the equation VE = 100% × (1 − odds ratio), determined from logistic regression models (*8*). The 95% CIs were calculated as 1 − CI_OR_, where CI_OR_ is the confidence interval of the odds ratio estimates. Models were adjusted a priori for suspected confounders, including U.S. Census region, calendar month, age (as a continuous variable), sex, and race/ethnicity. Other factors were included in the model if they changed the adjusted odds ratio of vaccination by >5%. Primary VE estimates were stratified by partial versus full vaccination. VE for patients reporting illness onset <14 days after receipt of the first dose of a 2-dose vaccine was also assessed. Because protective immunity is unlikely to be achieved immediately after vaccination (*4*,*5*,*7*), absence of VE within 14 days of the first dose was used as a proxy indicator of absence of bias in the primary VE estimates (*6*). Statistical analyses were conducted using SAS (version 9.4; SAS Institute). This activity was reviewed by CDC and the other participating institutions and was conducted consistent with applicable federal law and CDC policy.^††^

During January 1–March 26, 2021, 489 patients were eligible for participation, 72 (15%) of whom were excluded for the following reasons: 30 had SARS-CoV-2 testing >10 days after illness onset, 19 were hospitalized >14 days after illness onset, eight had onset of COVID-19–like illness after admission, three received the Janssen COVID-19 vaccine, and 12 had incomplete vaccination verification. Among the 417 patients included in the final analysis (including 187 case-patients and 230 controls), median age was 73 years for case-patients and controls, 48% were female, 17% were non-Hispanic Black, 6% were Hispanic (any race), 48% had one or more earlier hospitalizations in the last year, and 4% lived in a long-term care facility before admission (Table). Among the 187 case-patients, 19 (10%) had received at least 1 dose of Pfizer-BioNTech or Moderna vaccine ≥14 days before illness onset (including 18 [10%] who were partially vaccinated and one [0.5%] who was fully vaccinated) compared with 62 (27%) of 230 test-negative controls (including 44 [19%] and 18 [8%] who were partially and fully vaccinated, respectively). Prevalence of receipt of Pfizer-BioNTech and Moderna vaccines was similar (53% and 47%, respectively, among those vaccinated with ≥1 doses). Adjusted VE for full vaccination using Pfizer-BioNTech or Moderna vaccine was 94% (95% CI = 49%–99%), and adjusted VE for partial vaccination was 64% (95% CI = 28%–82%) (Figure). There was no significant effect for receiving the first dose of a 2-dose COVID-19 vaccine series within 14 days before illness onset (adjusted VE = 3%, 95% CI = −94%–51%).

**TABLE:** Characteristics of adults aged ≥65 years with COVID-19–like illness* tested for SARS-CoV-2 infection, by COVID-19 case status^†^ — 24 medical centers in 14 states,^§^ January–March 2021

Characteristic	Case status, no. (column %)			
	Total (N = 417)	Case-patients (n = 187)	Control participants (n = 230)	p-value
**Month of admission**				
January	80 (19)	52 (28)	28 (12)	<0.01
February	153 (37)	74 (40)	79 (34)	
March	184 (44)	61 (33)	123 (53)	
**U.S. Census region^¶^**				
Northeast	174 (42)	61 (33)	113 (49)	<0.01
South	135 (32)	77 (41)	58 (25)	
Midwest	68 (16)	23 (12)	45 (20)	
West	40 (10)	26 (14)	14 (6)	
**Age group, yrs**				
65–74	244 (59)	106 (57)	138 (60)	0.49
≥75	173 (41)	81 (43)	92 (40)	
**Female sex**	200 (48)	83 (44)	117 (51)	0.19
**Race/Ethnicity**				
White, non-Hispanic	303 (73)	129 (69)	174 (76)	0.32
Black, non-Hispanic	70 (17)	34 (18)	36 (16)	
Other, non-Hispanic	14 (3)	9 (5)	5 (2)	
Hispanic, any race	26 (6)	12 (6)	14 (6)	
Unknown	4 (1)	3 (2)	1 (0.4)	
**Medical insurance (missing = 1)**				
Yes	408 (98)	180 (96)	228 (99)	0.01
No	8 (2)	7 (4)	1 (0.4)	
Resident in long-term care facility** (missing = 1)	16 (4)	6 (3)	10 (4)	0.55
≥1 previous hospitalization in last year** (missing = 12)	195 (48)	63 (35)	132 (59)	<0.01
Received current season influenza vaccination** (missing = 18)	312 (78)	134 (76)	178 (80)	0.38
Current tobacco use** (missing = 8)				
Yes	35 (9)	8 (4)	27 (12)	<0.01
No	374 (91)	174 (96)	200 (88)	
**SARS-CoV-2 vaccination status^†^**				
Unvaccinated	287 (69)	146 (78)	141 (61)	<0.01
Single-dose vaccinated <14 days before illness onset	49 (12)	22 (12)	27 (12)	
Partially vaccinated	62 (15)	18 (10)	44 (19)	
Fully vaccinated	19 (5)	1 (0.5)	18 (8)	
**Vaccine type, if vaccinated (missing = 11)**				
Pfizer-BioNTech	63 (53)	15 (42)	48 (58)	0.10
Moderna	56 (47)	21 (58)	35 (42)	
**Admission characteristic**				
Days from illness onset to admission, median (IQR)	3 (1–6)	4 (1–7)	2 (0–4)	<0.01
Days from illness onset to SARS-CoV-2 testing, median (IQR)	2 (0–4)	3 (0–5)	1 (0–4)	<0.01

**Abbreviations:** HAIVEN = Hospitalized Adult Influenza Vaccine Effectiveness Network; IQR = interquartile range; IVY = Influenza and Other Viruses in the Acutely Ill.

* Clinical criteria for hospitalized COVID-19–like illness varied by hospital network. IVY Network criteria for COVID-19–like illness included presence of fever, feverishness, cough, sore throat, myalgias, shortness of breath, chest pain, loss of taste, loss of smell, respiratory congestion, increased sputum production, new oxygen saturation <94% on room air, new requirement for invasive or noninvasive mechanical ventilation, or new pulmonary findings on chest imaging consistent with pneumonia. HAIVEN criteria included fever without a known non–COVID-19 cause, new or worsening cough, a change in sputum production, or new or worsening shortness of breath.

**^†^** SARS-CoV-2 vaccination status included the following four categories: 1) unvaccinated, defined as no receipt of any SARS CoV-2 vaccine; 2) single-dose vaccinated <2 weeks before illness onset, defined as receipt of the first vaccine dose within 14 days before onset of COVID-like illness; 3) partially vaccinated, defined as receipt of 1 dose of a 2-dose vaccine series (Pfizer-BioNTech or Moderna) ≥14 days before illness onset or receipt of 2 doses, with the second dose received <14 days before illness onset; 4) fully vaccinated, defined as receipt of both doses of a 2-dose vaccine series, with the second dose received ≥14 days before illness onset.

**^§^** Patients were enrolled from 24 medical centers in 14 states (University of California Los Angeles and Stanford University [California], UCHealth University of Colorado Hospital [Colorado], Johns Hopkins Hospital [Maryland], Beth Israel Deaconess Medical Center and Baystate Medical Center [Massachusetts], University of Michigan, Henry Ford, and St. Joseph [Michigan], Hennepin County Medical Center [Minnesota], Montefiore Healthcare Center [New York], Wake Forest University [North Carolina], Ohio State University [Ohio], Oregon Health & Science University [Oregon], University of Pittsburgh Medical Center, Shadyside, Mercy, Passavant, St. Margaret, and Presbyterian Hospitals [Pennsylvania], Vanderbilt University Medical Center [Tennessee], Baylor Scott & White Medical Center, Temple, Round Rock, Hillcrest/Waco [Texas], and Intermountain Health [Utah]).

**^¶^** *Northeast*: Connecticut, Maine, Massachusetts, New Hampshire, New Jersey, New York, Pennsylvania, Rhode Island, and Vermont; *Midwest*: Illinois, Indiana, Iowa, Kansas, Michigan, Minnesota, Missouri, Nebraska, North Dakota, Ohio, South Dakota, and Wisconsin; *South*: Alabama, Arkansas, Delaware, District of Columbia, Florida, Georgia, Kentucky, Louisiana, Maryland, Mississippi, North Carolina, Oklahoma, South Carolina, Tennessee, Texas, Virginia, and West Virginia; *West*: Alaska, Arizona, California, Colorado, Hawaii, Idaho, Montana, Nevada, New Mexico, Oregon, Utah, Washington, and Wyoming.

** Information was obtained by patient or proxy self-report.

**FIGURE:**
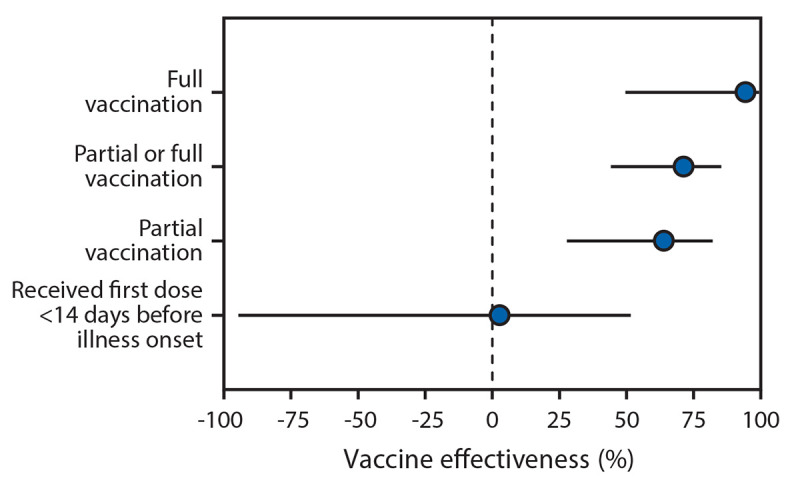
Adjusted* vaccine effectiveness (with 95% confidence intervals) against COVID-19 among hospitalized^†^ adults aged ≥65 years, by vaccination status^§^ — 24 medical centers in 14 states,^¶^ January–March 2021 **Abbreviations:** HAIVEN = Hospitalized Adult Influenza Vaccine Effectiveness Network; IVY = Influenza and Other Viruses in the Acutely Ill. * Vaccine effectiveness estimates were adjusted for U.S. Census region, calendar month, continuous age in years, sex, race and ethnicity (non-Hispanic White, non-Hispanic Black, non-Hispanic other or unknown, or Hispanic of any race), and one or more versus zero self-reported previous hospitalizations in the past year. ^†^ Clinical criteria for hospitalized COVID-19–like illness varied by hospital network. IVY Network criteria for COVID-19–like illness included presence of fever, feverishness, cough, sore throat, myalgias, shortness of breath, chest pain, loss of taste, loss of smell, respiratory congestion, increased sputum production, new oxygen saturation <94% on room air, new invasive or noninvasive ventilation, or new pulmonary findings on chest imaging consistent with pneumonia in the IVY Network; criteria included fever without a known non–COVID-19 cause, new or worsening cough, a change in sputum production, or new or worsening shortness of breath in the HAIVEN network. ^§^ SARS-CoV-2 vaccination status included the following four categories: 1) unvaccinated, defined as no receipt of any SARS CoV-2 vaccine; 2) first vaccine dose <14 days before illness onset, defined as a single dose of vaccine within 14 days prior to onset of COVID-19–like illness; 3) partially vaccinated, defined as receipt of 1 dose of a 2-dose vaccine series (Pfizer-BioNTech or Moderna) ≥14 days before illness onset or 2 doses with the second dose received <14 days before illness onset); 4) fully vaccinated, defined as receipt of both doses of a 2-dose vaccine series ≥14 days before illness onset. **^¶^** Patients were enrolled from 24 medical centers in 14 states (University of California Los Angeles and Stanford University [California], UCHealth University of Colorado Hospital [Colorado], Johns Hopkins Hospital [Maryland], Beth Israel Deaconess Medical Center and Baystate Medical Center [Massachusetts], University of Michigan, Henry Ford, and St. Joseph [Michigan], Hennepin County Medical Center [Minnesota], Montefiore Healthcare Center [New York], Wake Forest University [North Carolina], Ohio State University [Ohio], Oregon Health & Science University [Oregon], University of Pittsburgh Medical Center, Shadyside, Mercy, Passavant, St. Margaret, and Presbyterian Hospitals [Pennsylvania], Vanderbilt University Medical Center [Tennessee], Baylor Scott & White Medical Center, Temple, Round Rock, Hillcrest/Waco [Texas], and Intermountain Health [Utah]).

## Discussion

Monitoring the effectiveness of SARS-CoV-2 vaccination under routine public health use and specifically against severe outcomes in patients at higher risk, including older adults, is a high priority. In this multistate analysis of adults aged ≥65 years, receipt of an authorized COVID-19 vaccine was associated with significant protection against COVID-19 hospitalization. Effectiveness was 94% among adults who were fully vaccinated and 64% among adults who were partially vaccinated (i.e., onset of COVID-like illness ≥14 days after the first vaccine dose in a 2-dose series but <14 days after the second dose). These findings are consistent with efficacy determined from clinical trials in the subgroup of adults aged ≥65 years (*4*,*5*). Early reports from Israel have also documented the real-world effectiveness of SARS-CoV-2 vaccination, including among older adults (*7*,*9*). However, those postmarketing reports only represented the Pfizer-BioNTech vaccine. In the current report, Pfizer-BioNTech and Moderna vaccine products were equally represented, and approximately one half of the patients were aged ≥75 years, providing evidence of real-world effectiveness of both vaccines against an important measure of severe COVID-19 in older adults. Moreover, in assessing the impact of receiving only a single dose, no significant vaccine effectiveness <14 days after the first dose of a SARS-CoV-2 vaccine was detected. This suggests that bias is unlikely in the primary estimates of vaccine effectiveness from partial and full vaccination. This also highlights the continued risk for severe illness shortly after vaccination, before a protective immune response has been achieved and reinforces the need for vaccinated adults to continue physical distancing and prevention behaviors, such as use of face masks and recommended hand hygiene at least 14 days after the second dose of a 2-dose vaccine. The findings suggest that SARS-CoV-2 vaccines can reduce the risk for COVID-19–associated hospitalization and, as a consequence of preventing severe COVID-19, vaccination might have an impact on post-COVID conditions (e.g., “long COVID”) and deaths (*2*,*10*).

The findings in this report are subject to at least six limitations. First, the CIs for VE estimates were wide because of the small sample size, and the number of participants was too small to assess VE by vaccine product, age group, or underlying conditions. Second, as an interim analysis that included self-reported data, vaccination status might have been misclassified, or participants might have had imperfect recollection of vaccination or illness onset dates. Third, selection bias and residual confounding cannot be excluded. Fourth, although the analysis included hospitalized adults from 14 states, the participants were not geographically representative of the U.S. population. Fifth, the case-control design infers protection based on associations between disease outcome and previous vaccination but cannot establish causation. Finally, duration of VE and VE for nonhospitalized COVID-19 was not assessed.

During January–March 2021, in a multistate network of U.S. hospitals, vaccination was associated with a reduced risk for COVID-19–associated hospitalization among adults aged ≥65 years. These data suggest that continuing to rapidly vaccinate U.S. adults against COVID-19 will likely have a marked impact on COVID-19 hospitalization and might lead to commensurate reductions in post-COVID conditions and deaths (*2*,*10*).

**Summary**

**What is already known about this topic?**

Clinical trials suggest high efficacy for COVID-19 vaccines, but evaluation of vaccine effectiveness against severe outcomes in real-world settings and in populations at high risk, including older adults, is needed.

**What is added by this report?**

In a multistate network of U.S. hospitals during January–March 2021, receipt of Pfizer-BioNTech or Moderna COVID-19 vaccines was 94% effective against COVID-19 hospitalization among fully vaccinated adults and 64% effective among partially vaccinated adults aged ≥65 years.

**What are the implications for public health practice?**

SARS-CoV-2 vaccines significantly reduce the risk for COVID-19–associated hospitalization in older adults and, in turn, might lead to commensurate reductions in post-COVID conditions and deaths.

## Footnotes

* Patients were enrolled from 24 medical centers in 14 states (University of California Los Angeles and Stanford University [California], UCHealth University of Colorado Hospital [Colorado], Johns Hopkins Hospital [Maryland], Beth Israel Deaconess Medical Center and Baystate Medical Center [Massachusetts], University of Michigan, Henry Ford, and St. Joseph [Michigan], Hennepin County Medical Center [Minnesota], Montefiore Healthcare Center [New York], Wake Forest University [North Carolina], Ohio State University [Ohio], Oregon Health & Science University [Oregon], University of Pittsburgh Medical Center, Shadyside, Mercy, Passavant, St. Margaret, and Presbyterian Hospitals [Pennsylvania], Vanderbilt University Medical Center [Tennessee], Baylor Scott & White Medical Center, Temple, Round Rock, Hillcrest/Waco [Texas], and Intermountain Health [Utah]).

† Partially vaccinated is defined as receipt of 1 dose of a 2-dose vaccine series (Pfizer-BioNTech or Moderna vaccines) ≥14 days before illness onset or 2 doses with the second dose received <14 days before illness onset. Fully vaccinated is defined as receipt of both doses of a 2-dose vaccine series, with the second dose received ≥14 days before illness onset.

§ Pfizer-BioNTech and Moderna COVID-19 vaccines are approved for use under an EUA in the United States. The Vaccine Adverse Event Reporting System (VAERS) is used to detect possible signals of adverse events associated with vaccines. Adverse events related to these COVID-19 vaccines can be reported at https://www.fda.gov/vaccines-blood-biologics/report-problem-center-biologics-evaluation-research/vaccine-adverse-events or https://vaers.hhs.gov/reportevent.html.

¶ IVY Network criteria for COVID-19–like illness included presence of fever, feverishness, cough, sore throat, myalgias, shortness of breath, chest pain, loss of taste, loss of smell, respiratory congestion, increased sputum production, new oxygen saturation <94% on room air, new requirement for invasive or noninvasive mechanical ventilation, or new pulmonary findings on chest imaging consistent with pneumonia. HAIVEN criteria included fever without a known non–COVID-19 cause, new or worsening cough, a change in sputum production, or new or worsening shortness of breath.

** Based on postmarketing findings from Israel, where VE was observed at 14 days after vaccination after 1 dose.

†† 45 C.F.R. part 46.102(l)(2), 21 C.F.R. part 56; 42 U.S.C. Sect. 241(d); 5 U.S.C. Sect. 552a; 44 U.S.C. Sect. 3501 et seq.

## References

[1] Garg S, Kim L, Whitaker M, Hospitalization rates and characteristics of patients hospitalized with laboratory-confirmed coronavirus disease 2019—COVID-NET, 14 states, March 1–30, 2020. MMWR Morb Mortal Wkly Rep 2020;69:458–64. 10.15585/mmwr.mm6915e3 32298251 PMC7755063

[2] Wortham JM, Lee JT, Althomsons S, Characteristics of persons who died with COVID-19—United States, February 12–May 18, 2020. MMWR Morb Mortal Wkly Rep 2020;69:923–9. 10.15585/mmwr.mm6928e1 32673298

[3] Dooling K, Marin M, Wallace M, The Advisory Committee on Immunization Practices’ updated interim recommendation for allocation of COVID-19 vaccine—United States, December 2020. MMWR Morb Mortal Wkly Rep 2021;69:1657–60. 10.15585/mmwr.mm695152e2 33382671 PMC9191902

[4] Baden LR, El Sahly HM, Essink B, ; COVE Study Group. Efficacy and safety of the mRNA-1273 SARS-CoV-2 vaccine. N Engl J Med 2021;384:403–16. 10.1056/NEJMoa2035389 33378609 PMC7787219

[5] Polack FP, Thomas SJ, Kitchin N, ; C4591001 Clinical Trial Group. Safety and efficacy of the BNT162b2 mRNA Covid-19 vaccine. N Engl J Med 2020;383:2603–15. 10.1056/NEJMoa2034577 33301246 PMC7745181

[6] Patel MM, Jackson ML, Ferdinands J. Postlicensure evaluation of COVID-19 vaccines. JAMA 2020;324:1939–40. 10.1001/jama.2020.19328 33064144

[7] Dagan N, Barda N, Kepten E, BNT162b2 mRNA Covid-19 vaccine in a nationwide mass vaccination setting. N Engl J Med 2021;384:1412–23. 10.1056/NEJMoa2101765 33626250 PMC7944975

[8] Jackson ML, Nelson JC. The test-negative design for estimating influenza vaccine effectiveness. Vaccine 2013;31:2165–8. 10.1016/j.vaccine.2013.02.053 23499601

[9] Rinott E, Youngster I, Lewis YE. Reduction in COVID-19 patients requiring mechanical ventilation following implementation of a national COVID-19 vaccination program—Israel, December 2020–February 2021. MMWR Morb Mortal Wkly Rep 2021;70:326–8. 10.15585/mmwr.mm7009e3 33661863 PMC7948930

[10] Huang C, Huang L, Wang Y, 6-month consequences of COVID-19 in patients discharged from hospital: a cohort study. Lancet 2021;397:220–32. 10.1016/S0140-6736(20)32656-8 33428867 PMC7833295

